# O4.4. DOES POLYGENIC RISK SCORE FOR SCHIZOPHRENIA MODERATE THE MOMENTARY AFFECTIVE AND PSYCHOTIC REACTIONS TO DAILY-LIFE STRESSORS?

**DOI:** 10.1093/schbul/sby015.210

**Published:** 2018-04-01

**Authors:** Lotta-Katrin Pries, Sinan Guloksuz, Claudia Menne-Lothmann, Jeroen Decoster, Ruud van Winkel, Dina Collip, Philippe Delespaul, Marc De Hert, Catherine Derom, Evert Thiery, Nele Jacobs, Marieke Wichers, Boris Klingenberg, Ozan Cinar, Bochao Lin, Jurjen Luykx, Bart Rutten, Jim van Os

**Affiliations:** 1 Maastricht University Medical Centre; 2 Maastricht University Medical Centre, Yale School of Medicine; 3 Maastricht University Medical Centre, University Psychiatric Centre KU Leuven; 4 University Psychiatric Centre KU Leuven; 5 Centre of Human Genetics, University Hospitals Leuven, Ghent University Hospitals, Ghent University; 6 Ghent University Hospital; 7 Maastricht University Medical Centre, Open University of the Netherlands; 8 Maastricht University Medical Centre, University of Groningen, University Medical Center Groningen, Interdisciplinary Center Psychopathology and Emotion Regulation; 9 Brain Center Rudolf Magnus, University Medical Center Utrecht; 10 Maastricht University Medical Centre, Brain Center Rudolf Magnus, University Medical Center Utrecht, King’s College London, King’s Health Partners, Institute of Psychiatry

## Abstract

**Background:**

Studies using the event sampling method (ESM), a structured diary technique measuring subjective experiences and emotional fluctuations in daily life, have consistently shown that individuals reporting psychotic experiences display a heightened emotional reactivity to minor stressors—a neuropsychological mechanism that likely contributes to the development and perpetuation of psychotic experiences. Except a few undersized non-replicated candidate-gene studies showing an association between genetic variations and elevated momentary stress reactivity, genetic underpinnings of emotion reactivity to momentary stressors have not been investigated. Therefore, by leveraging a large general population twin dataset of ESM, we aimed to investigate—for the first time—whether the polygenic risk score (PRS) for schizophrenia moderates stress reactivity (psychotic experiences (PE) and negative affect (NA) in response to momentary stress).

**Methods:**

Data were derived from a general population adolescent and young adult twin sample. The total sample included 638 participants (Monozygotic = 202, Dizygotic = 436). ESM variables were randomly measured at 10 times/day over 6 consecutive days. For the main analyses, we assessed ESM information on PE (suspiciousness, loss of control, racing thoughts, pervasive thoughts, difficulties to express thoughts), NA (feeling lonely, anxious, listless, down, guilty), and event-related stress (pleasantness of the most important event since last entry); and for additional explorative analyses we assessed social stress (participants were asked with whom they are (e.g. nobody or family) and to rate the pleasantness of the social situation). PRS were trained on the results from the Psychiatric Genetics Consortium-2 SZ. Multilevel regression analyses, taking into account of multiple observations nested within twins who were clustered within family, were used to analyze the moderating effects of PRS (at p-value < 0.05) on the relationship between momentary stress and NA or PE. All analyses were adjusted for age, sex and 2 principle components.

**Results:**

There were significant main effects of momentary stress (event stress: b = 0.065, p < 0.001, 95% CI = 0.048, 0.082; social stress: b = 0.128; p < 0.001; 95% CI = 0.111, 0.145) on PE. However, neither the main effects of PRS on PE nor the interaction between PRS and momentary stress on PE were significant. The analysis with NA as dependent variable indicated main effects of momentary stress (event stress: b = 0.096; p <0.001; 95% CI = 0.081, 0.111; social stress: b = 0.180; p < 0.001; 95% CI = 0.164, 0.197), but no main effect of PRS. There was a significant negative interaction between PRS and both event-related stress (b = -0.016; p = 0.024; 95% CI = -0.031, -0.002) and social stress (b = -0.017; p = 0.024; 95% CI = -0.031, -0.002) on NA.

**Discussion:**

This is the first study investigating the influence of molecular genetic risk for schizophrenia on momentary stress reactivity, measured using an ecologically valid diary method. These results suggest that PRS for schizophrenia does not have an effect on psychotic stress responses, while increased genetic risk for schizophrenia showed a buffering effect on the association between momentary stress and NA. It is possible that individuals with high PRS for schizophrenia might have emotional response deficits, a characteristic of the clinical phenotype. Alternatively, these individuals might have been less accurate in self-evaluating momentary stress, attributing high values to stressors that genuinely do not have an impact on their emotion regulation. Future studies, investigating both clinical and general populations, are required to elucidate the impact of PRS on stress reactivity.

